# Tirofiban combined with aspirin for branch atheromatous disease: a propensity score–matched study

**DOI:** 10.3389/fneur.2026.1802990

**Published:** 2026-04-01

**Authors:** Xiuxiu Lu, Liqiong Cao, Li Zeng, Liangbing Zhang

**Affiliations:** Department of Neurology, The First People’s Hospital of Anqing Affiliated to Anhui Medical University, Anqing, China

**Keywords:** aspirin, branch atheromatous disease, dual antiplatelet therapy, early neurological deterioration, propensity score matching, tirofiban

## Abstract

**Background:**

The optimal antiplatelet approach for branch atheromatous disease (BAD) remains uncertain. We evaluated whether early administration of tirofiban plus aspirin (T + A) improves outcomes compared with dual antiplatelet therapy (DAPT) and assessed its potential as rescue therapy for early neurological deterioration (END).

**Methods:**

This single-center retrospective cohort study included patients with acute BAD treated between November 2022 and August 2025. Patients received either T + A or DAPT. Propensity score matching (caliper 0.02) was performed to balance baseline characteristics. Primary outcomes were END within 7 days and excellent functional outcome (mRS 0–1) at 90 days; secondary outcomes included favorable outcome (mRS 0–2) and early neurological improvement.

**Results:**

After matching, END occurred less frequently in the T + A group than in the DAPT group (10.1% vs. 55.1%; adjusted OR 0.08; 95% CI 0.03–0.20; *p* < 0.001). Excellent functional outcome at 90 days was more common with T + A (88.4% vs. 58.0%; adjusted OR 5.17; 95% CI 2.12–12.61; *p* < 0.001). T + A also improved early neurological recovery and favorable functional outcomes. Among patients who deteriorated on DAPT, exploratory rescue T + A was associated with improved recovery. No major bleeding increase was observed. These results are specific to this single-center cohort of Chinese patients with acute, mild-to-moderate BAD (NIHSS <15).

**Conclusion:**

In this single-center cohort of Chinese patients with acute, mild-to-moderate BAD (NIHSS <15), tirofiban plus aspirin reduced END and improved 90-day functional outcomes. Exploratory evidence suggests it may also serve as a rescue therapy for patients deteriorating under DAPT. However, its efficacy and safety require validation in prospective, randomized studies.

## Introduction

Branch atheromatous disease (BAD) represents a distinct and clinically important subtype of ischemic stroke and is reported more frequently in East Asian populations than in Western cohorts (1). Pathologically, BAD is characterized by atherosclerotic plaques in intracranial parent arteries that extend into the origins of perforating branches. This process exposes the perforator ostia to abnormal shear stress, facilitates platelet activation, and promotes progressive thrombus formation, ultimately leading to the characteristic elongated or columnar infarction pattern on imaging (2).

From a clinical perspective, BAD is notable for its propensity toward early neurological deterioration (END). Previous studies have reported END rates of up to 38.1% (3), with short-term disability [modified Rankin Scale (mRS) ≥ 3] occurring in as many as 61% of patients (4). Such early worsening is strongly associated with unfavorable long-term functional outcomes, underscoring the need for effective early management strategies.

Dual antiplatelet therapy (DAPT) is currently the most commonly adopted antithrombotic regimen for patients with BAD (5). However, its ability to prevent or halt END appears limited. Observational data indicate that END may still occur in up to 34.5% of patients within 48 h despite DAPT, and approximately 38.2% remain disabled at 90 days (6). Intravenous thrombolysis offers only modest benefit in this population, largely due to narrow time windows and a high risk of early reocclusion (7, 8). Likewise, intensified strategies such as the combination of argatroban with DAPT have not consistently translated into meaningful functional improvement, with END rates remaining substantial (9, 10). Collectively, these findings suggest that currently available antithrombotic approaches do not adequately address the pathophysiological mechanisms driving deterioration in BAD.

Tirofiban, a fast-acting and reversible glycoprotein IIb/IIIa receptor antagonist, has attracted increasing attention in the setting of acute ischemic stroke. Previous studies have shown that tirofiban can reduce the incidence of END and improve functional outcomes in patients with non-cardioembolic stroke, particularly among those who are not candidates for reperfusion therapy (11–13). Moreover, tirofiban has been explored as a rescue treatment for neurological worsening, with trials such as RESCUE BT2 reporting improved neurological recovery and higher rates of favorable functional outcomes at 90 days (14–16).

Despite these encouraging observations, evidence specifically addressing the role of tirofiban in BAD remains sparse. In particular, it is unclear whether tirofiban combined with aspirin offers superior protection against END compared with standard DAPT, or whether tirofiban can serve as an effective rescue option after DAPT failure in patients with BAD. To address these gaps, we conducted a propensity score–matched cohort study to compare the efficacy and safety of tirofiban plus aspirin versus DAPT in acute BAD and to explore the potential rescue value of tirofiban in BAD-related END.

## Methods

### Study design and population

This was a single-center retrospective cohort study conducted at the First People’s Hospital of Anqing affiliated to Anhui Medical University. Adult patients aged 18–80 years who were admitted with acute ischemic stroke between November 2022 and August 2025 were retrospectively identified from the institutional stroke database. During the study period, antiplatelet treatment strategies followed routine clinical practice and were determined by treating neurologists according to patients’ clinical characteristics and imaging findings.

Patients were categorized into the tirofiban plus aspirin (T + A) group or the dual antiplatelet therapy (DAPT) group based on the initial antiplatelet regimen initiated within 48 h of symptom onset. The study protocol was approved by the Ethics Committee of the First People’s Hospital of Anqing affiliated to Anhui Medical University (No. AQYY–YXLL–LWLL–56). Given the retrospective design and anonymized data collection, the requirement for informed consent was waived.

Patient selection and grouping.

Eligible patients presented within 48 h of symptom onset and met established clinical and imaging criteria for branch atheromatous disease (BAD). BAD was defined as a single acute infarction within the territory of a perforating artery on diffusion-weighted imaging (DWI), with longitudinal extension along the perforator and maximal lesion diameter ≥15 mm, consistent with classical descriptions (3, 17, 18).

Under our MRI acquisition parameters (3.0-T scanner; slice thickness 5 mm; interslice gap 1 mm), lesions spanning ≥2 consecutive axial slices were considered to reliably reflect longitudinal perforator extension. All DWI images were independently reviewed by two experienced stroke neurologists, with discrepancies resolved by consensus. Parent artery pathology was evaluated using magnetic resonance angiography and/or computed tomography angiography; patients with ≥70% stenosis or complete occlusion were excluded to minimize inclusion of large-artery infarctions. Additional inclusion criteria included baseline NIHSS <15. Patients were excluded for contraindications to antiplatelet therapy, prior dual antiplatelet or anticoagulant therapy, severe hepatic or renal dysfunction, pre-stroke disability (mRS ≥ 2), or prior intravenous thrombolysis/endovascular treatment. Patients receiving rt-PA were excluded to avoid confounding from thrombolysis-related early neurological changes and variable timing of antiplatelet initiation (5, 19).

### Propensity score matching

To reduce baseline imbalance related to non-random treatment allocation, propensity score matching (PSM) was performed in a 1:1 ratio. Variables included in the propensity score model were selected *a priori* based on clinical relevance and previously reported associations with treatment choice and early neurological deterioration (20). These variables included age, sex, baseline NIHSS score, baseline mRS score, and onset-to-treatment time.

Propensity scores were estimated using multivariable logistic regression to model the probability of receiving tirofiban plus aspirin versus DAPT. Nearest-neighbor matching without replacement was conducted using a caliper width of 0.02 of the standard deviation of the logit of the propensity score. Covariate balance before and after matching was assessed using standardized mean differences, with values <0.10 indicating adequate balance. In addition, kernel density plots of propensity scores before and after matching were generated (Supplementary Figure 1), showing substantial overlap between groups after matching and confirming successful balance and common support. All primary efficacy and safety analyses were performed in the matched cohort.

Inflammatory biomarkers were not incorporated into the propensity score model to avoid potential overadjustment, as these variables may represent intermediate factors on the causal pathway between antiplatelet treatment and the development of early neurological deterioration.

### Treatment regimens

#### Tirofiban plus aspirin (T + a) group

Patients received intravenous tirofiban administered as a loading dose of 0.4 μg/kg/min for 30 min, followed by a maintenance infusion of 0.1 μg/kg/min for 48 h, in combination with oral aspirin (100 mg once daily). The 48-h infusion duration was chosen to cover the high-risk period for END in BAD (typically 24–72 h after symptom onset), ensuring continuous antiplatelet coverage during this critical phase of thrombus propagation. This regimen leverages tirofiban’s rapid onset and short half-life to provide potent, reversible platelet inhibition during the acute unstable phase, and its duration is consistent with protocols established in previous studies of tirofiban in acute ischemic stroke (21–23). Four hours before stopping tirofiban, clopidogrel (75 mg daily) was initiated to transition to a 21-day dual antiplatelet course, followed by long-term monotherapy with aspirin or clopidogrel per physician discretion.

#### Dual antiplatelet therapy group

Patients received standard dual antiplatelet therapy consisting of aspirin (100 mg once daily) and clopidogrel (75 mg once daily) for 21 days, followed by long-term antiplatelet monotherapy.

During the study period, clopidogrel was initiated at a maintenance dose of 75 mg daily without routine loading, reflecting institutional practice for BAD management.

Among patients initially treated with DAPT who developed early neurological deterioration, escalation to tirofiban infusion was considered after individualized assessment of bleeding risk. These patients were classified as the rescue therapy subgroup. Patients who continued DAPT without escalation constituted the continued DAPT subgroup. The rescue therapy analysis was defined *post hoc* to explore the potential role of tirofiban as a salvage strategy for BAD-related early neurological deterioration. This post-hoc analysis was exploratory in nature and subject to potential indication and immortal time bias; therefore, the results should be interpreted with caution.

### Outcomes

The primary efficacy outcomes were the occurrence of early neurological deterioration (END) within 7 days of symptom onset and excellent functional outcome, defined as an mRS score of 0–1 at 90 days (11). END was defined as an increase of ≥2 points in the total NIHSS score or an increase of ≥1 point in the motor subscore within 7 days and was considered the principal short-term indicator of early treatment failure in BAD (24).

Secondary outcomes included a favorable functional outcome (mRS score 0–2) at 90 days (11), early neurological improvement (ENI), defined as a decrease of ≥2 points in the NIHSS score within 7 days (10), and the ordinal distribution of mRS scores at 90 days. Changes in NIHSS scores over time and mRS scores at baseline and 90 days were also evaluated.

Safety outcomes included symptomatic intracranial hemorrhage (sICH) within 72 h, stroke recurrence, and any hemorrhage within 90 days. sICH was defined as intracranial bleeding on CT or MRI accompanied by neurological deterioration, indicated by a ≥ 4-point increase in NIHSS from baseline, according to the ECASS II criteria (25). Asymptomatic hemorrhages, such as small punctate or microbleeds on imaging without clinical worsening, were recorded as overall bleeding events. All patients were followed for 90 days.

### Statistical analysis

All primary analyses were conducted in the propensity score–matched cohort.

Binary outcomes (END, mRS 0–1, mRS 0–2, ENI) were analyzed using generalized linear models with a logit link function (i.e., logistic regression) implemented in SPSS (version 27.0). In the matched cohort, multivariable logistic regression was performed to adjust for residual imbalance. The adjusted model included the TyG index, which remained imbalanced after propensity score matching; other matching covariates (age, sex, baseline NIHSS score, baseline mRS score, and onset-to-treatment time) were well balanced and not included in the adjusted model. Effect estimates were reported as odds ratios (ORs) with 95% confidence intervals (CIs). Risk ratios and risk differences were calculated descriptively using standard formulas in SPSS to facilitate clinical interpretation.

Ordinal outcomes (distribution of 90-day mRS scores) were analyzed using proportional odds models (ordinal logistic regression) in SPSS (version 27.0).

Changes in NIHSS scores over time were analyzed using rank-transformed covariance analysis to account for non-normal distributions. This analysis was performed in R software (version 4.5.1) using the afex package, and partial eta-squared [*η* (2)] was reported as the effect size.

Propensity score matching (PSM) was performed using the MatchIt package (version 4.5.1) in R (version 4.5.1). Nearest-neighbor 1:1 matching without replacement was conducted with a caliper width of 0.02 of the standard deviation of the logit of the propensity score. Covariate balance before and after matching was assessed using standardized mean differences.

Sensitivity analyses were conducted to assess the robustness of PSM by varying the caliper width (0.01 and 0.03) and adopting a 1:2 matching ratio using the MatchIt package in R. For each alternative matching approach, the main outcomes (END within 7 days and excellent functional outcome at 90 days) were analyzed using the same univariable and multivariable logistic regression approach described above, performed in SPSS. Furthermore, to evaluate the potential impact of inflammatory burden on treatment allocation, an additional sensitivity analysis was performed incorporating ln(SII) into the propensity score model. Covariate balance before and after matching for this analysis is presented in Supplementary Table S1, and the corresponding outcome analyses are summarized in Supplementary Table S2. This approach allowed us to preliminarily validate the role of systemic inflammation as a potential intermediate variable, confirming that inclusion of ln(SII) did not materially alter the estimated treatment effects.

The rescue therapy analysis was performed as a post-hoc exploratory analysis. Given the limited sample size and low event rates, Firth’s penalized logistic regression was applied using the logistf package in R (version 4.5.1) to mitigate small-sample bias and separation issues when estimating the association between rescue tirofiban therapy and 90-day functional outcomes.

Prespecified subgroup analyses were conducted to assess the consistency of treatment effects across clinically relevant strata, including age, onset-to-treatment time, baseline NIHSS score, and infarct location. These analyses were performed using binary logistic regression in SPSS (version 27.0), and interaction terms were tested to evaluate heterogeneity of treatment effects.

All statistical analyses were conducted using R software (version 4.5.1) with the MatchIt, logistf, and afex packages, and IBM SPSS Statistics (version 27.0) for generalized linear and ordinal logistic regression analyses. All tests were two-sided, and a *p* < 0.05 was considered statistically significant. To account for multiple testing across outcome indicators, false discovery rate (FDR) correction using the Benjamini–Hochberg method was applied, and FDR-adjusted *p*-values were reported.

## Results

### Patient cohort

**Figure 1:**
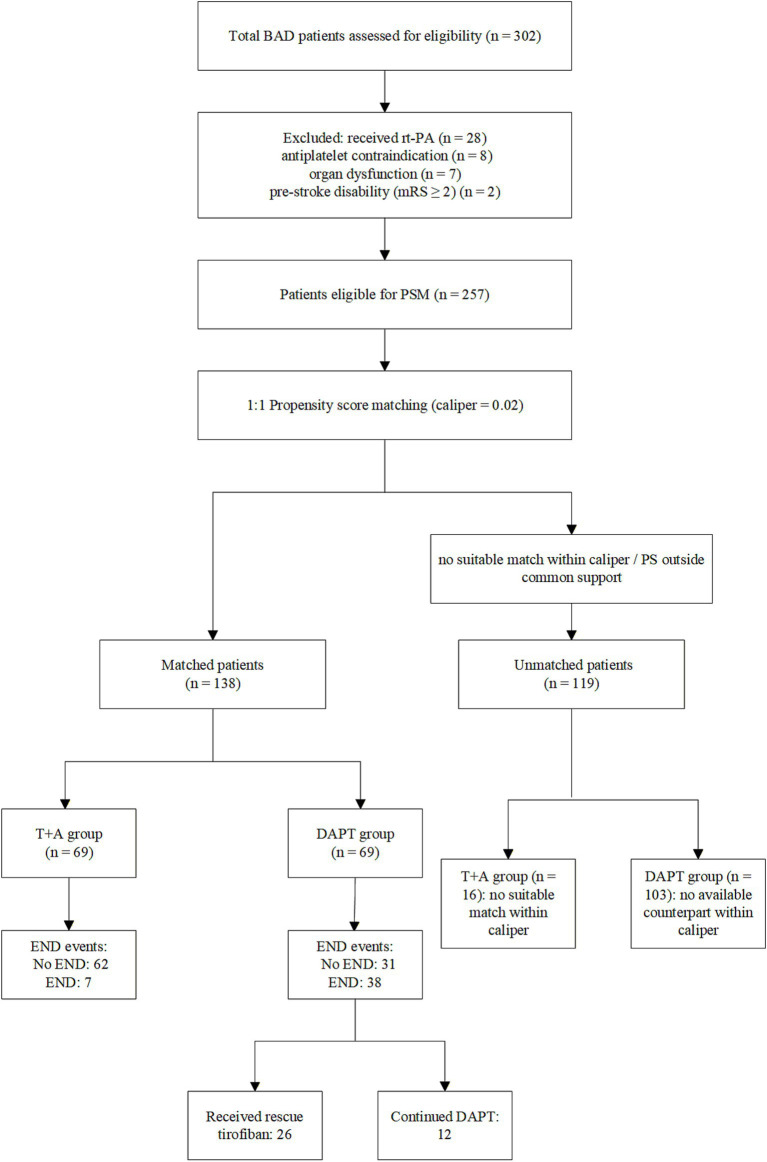
Flow diagram of patient screening, eligibility assessment, propensity score matching, and treatment allocation. Patients with branch atheromatous disease (BAD) were screened and assessed for eligibility. After applying the exclusion criteria, eligible patients underwent 1:1 propensity score matching (caliper = 0.02) to generate a matched cohort. Patients were then stratified according to the initial antiplatelet regimen: tirofiban plus aspirin (T + A) or dual antiplatelet therapy (DAPT). Patients without a suitable match within the caliper or outside the region of common support were classified as unmatched. Among patients initially treated with DAPT who developed early neurological deterioration (END), subsequent management included either rescue tirofiban therapy or continuation of DAPT.

A total of 257 patients met the predefined eligibility criteria, of whom 138 were successfully included in the propensity score–matched cohort (69 per group), while 119 patients were not successfully matched (T + A: *n* = 16; DAPT: *n* = 103). Baseline characteristics of the unmatched patients are summarized in Supplementary Table S3. To characterize the differences between included and excluded patients, Supplementary Table S4 presents the baseline characteristics of the matched and unmatched cohorts along with standardized mean differences (SMDs). These differences reflect the inherent selection process during propensity score matching and delineate the population to which our findings are most directly applicable. Within the matched cohort, early neurological deterioration occurred in 38 patients (55.1%) in the DAPT group. Of these, 26 patients subsequently received tirofiban as rescue therapy. The patient selection process and treatment allocation are illustrated in Figure 1.

Before propensity score matching, several baseline characteristics differed between the two treatment groups. Patients in the tirofiban plus aspirin group presented with higher baseline NIHSS and mRS scores and a shorter onset-to-treatment time compared with those in the DAPT group. In addition, inflammatory and metabolic indicators, including ln(SII), the triglyceride–glucose (TyG) index, and total cholesterol levels, were significantly higher in the tirofiban plus aspirin group. Other demographic variables, vascular risk factors, infarct locations, and imaging features were generally comparable between groups (Supplementary Table S5).

After propensity score matching, baseline balance between the two groups was markedly improved. All standardized mean differences for variables included in the matching model were reduced to below 0.10, indicating satisfactory covariate balance. Baseline characteristics of the matched cohort are presented in Table 1. Following matching, the TyG index remained imbalanced between groups (SMD = 0.46), with higher values observed in the tirofiban plus aspirin group, although the clinical significance of this difference remains uncertain. No other clinically meaningful differences were observed between groups.

**Table 1:** Baseline characteristics of the propensity score–matched cohort.

Characteristics	T + A (*n* = 69)	DAPT (*n* = 69)	*p*	SMD before	SMD after
Variables used for PSM					
Age, y; median (IQR)	60 (56, 72)	63 (58, 71)	0.600	0.158	0.057
Male, n (%)	47 (68.1)	48 (69.6)	0.854	0.293	0.031
Baseline NIHSS score, median (IQR)	4 (3, 5)	4 (3, 6)	0.779	0.429	0.046
Baseline mRS score, median (IQR)	3 (2, 4)	3 (2, 4)	0.869	0.459	0.050
Onset-to-treatment time, h; median (IQR)	10 (6, 24)	10 (6.4, 24)	0.587	0.507	0.028
Other clinical characteristics					
Hypertension, n (%)	50 (72.5)	46 (66.7)	0.459	0.008	0.126
Diabetes, n (%)	14 (20.3)	12 (17.4)	0.663	0.059	0.074
Coronary artery disease, n (%)	1 (1.4)	4 (5.8)	0.158	0.217	0.234
History of stroke, n (%)	6 (8.7)	10 (14.5)	0.288	0.075	0.182
Smoking, n (%)	31 (44.9)	33 (47.8)	0.733	0.241	0.058
Drinking, n (%)	23 (33.3)	17 (24.6)	0.260	0.231	0.193
Infarct location, n (%)			0.217	0.123	0.119
Anterior circulation	40 (58.0)	47 (68.1)			
Posterior circulation	29 (42.0)	22 (31.9)			
Maximum infarct area, mm^2^; median (IQR)	62 (39, 101)	72 (40, 110)	0.533	0.022	0.125
Maximum infarct diameter, mm; median (IQR)	11 (8, 15)	11 (7.2, 15)	0.638	0.074	0.009
Number of involved slices; median (IQR)	2 (2, 3)	2 (2, 3)	0.514	0.062	0.102
Systolic BP at admission, mmHg; mean ± SD	155.87 ± 23.19	156.10 ± 21.70	0.952	0.097	0.010
Diastolic BP at admission, mmHg; median (IQR)	100 (85, 102)	92 (80, 102)	0.484	0.146	0.131
Laboratory examination					
Ln(SII); median (IQR)	6.33 (6.10, 6.78)	6.15 (5.88, 6.74)	0.324	0.352	0.082
TyG index; mean ± SD	1.85 ± 0.79	1.52 ± 0.66	0.016	0.450	0.460
TC, mmol/L; mean ± SD	4.94 ± 1.09	4.80 ± 1.09	0.378	0.006	0.133
HDL, mmol/L; median (IQR)	1.10 (1.10, 1.12)	1.30 (1.05, 1.43)	0.831	0.134	0.137
LDL, mmol/L; median (IQR)	3.38 (2.86, 3.55)	3.07 (2.49, 3.50)	0.319	0.191	0.134

Values are presented as n (%), mean ± SD, or median (interquartile range), as appropriate. Propensity score matching was performed using a 1:1 nearest-neighbor method. Covariate balance after matching was primarily assessed using standardized mean differences (SMDs), with values <0.10 indicating adequate balance. *P*-values were calculated for descriptive purposes using the Student’s *t*-test or Mann–Whitney U test for continuous variables and the χ^2^ test or Fisher’s exact test for categorical variables, as appropriate. DAPT, dual antiplatelet therapy; HDL-C, high-density lipoprotein cholesterol; LDL-C, low-density lipoprotein cholesterol; mRS, modified Rankin Scale; NIHSS, National Institutes of Health Stroke Scale; SII, systemic immune-inflammation index; TyG, triglyceride–glucose index.

### Outcomes in the overall cohort

In the overall cohort, patients treated with tirofiban plus aspirin experienced a significantly lower incidence of early neurological deterioration compared with those receiving DAPT. In addition, higher rates of favorable functional outcomes at 90 days (mRS 0–1 and mRS 0–2) and early neurological improvement were observed in the tirofiban plus aspirin group. Despite the presence of baseline imbalances prior to matching, the direction and magnitude of treatment effects in the overall cohort were consistent with those observed after propensity score matching (Supplementary Table S6).

Exploratory multivariable analyses in the overall cohort showed that ln(SII) was independently associated with the occurrence of END. These analyses were hypothesis-generating in nature and were not adjusted for multiple testing. Treatment with tirofiban plus aspirin remained independently associated with a reduced risk of END in these models (Supplementary Table S7).

### Primary outcomes

Within 7 days of treatment initiation, early neurological deterioration occurred in 10.1% of patients in the tirofiban plus aspirin group, compared with 55.1% in the DAPT group. The unadjusted odds ratio for END was 0.09 (95% CI, 0.04–0.23; *p* < 0.001). After adjustment for prespecified covariates, tirofiban plus aspirin remained strongly associated with a lower risk of END (adjusted OR, 0.08; 95% CI, 0.03–0.20; *p* < 0.001).

At 90 days, excellent functional outcomes (mRS 0–1) were achieved by 88.4% of patients in the tirofiban plus aspirin group and 58.0% of patients in the DAPT group. After covariate adjustment, treatment with tirofiban plus aspirin was associated with a significantly higher likelihood of achieving an excellent outcome (adjusted OR, 5.17; 95% CI, 2.12–12.61; *p* < 0.001) (Table 2).

**Table 2:** Clinical outcomes after propensity score matching.

Outcomes	T + A (*n* = 69)	DAPT (*n* = 69)	Risk difference (95% CI)	Risk ratio (95% CI)	Unadjusted		Adjusted		FDR-adjusted P
					OR (95%CI)	*P*	OR (95%CI)	*P*	
Primary outcomes									
Early neurological deterioration (END), n (%)	7 (10.1)	38 (55.1)	−45.0 (−58.62 to −31.24)	0.18 (0.09–0.38)	0.09 (0.04–0.23)	<0.001	0.08 (0.03–0.20)	<0.001	0.001
Excellent functional outcome (mRS score 0–1) at 90 days	61 (88.40)	40 (58.00)	30.44 (16.56–44.31)	1.53 (1.23–1.90)	5.53 (2.30–13.31)	<0.001	5.17 (2.12–12.61)	<0.001	0.001
Secondary outcomes									
mRS score distribution at 90 d, median (IQR)	0 (0,1)	1 (1,2)	N/A	N/A	0.13 (0.07–0.27)	<0.001	0.14 (0.07–0.29)	<0.001	0.001
Favorable functional outcome (mRS score 0–2) at 90 days	67 (97.1)	58 (84.1)	13.04 (3.55–22.54)	1.16 (1.04–1.29)	6.35 (1.35–29.85)	0.019	6.50 (1.34–31.45)	0.020	0.020
Early neurological improvement (ENI)	52 (75.4)	25 (36.2)	39.13 (23.90–54.36)	2.08 (1.48–2.92)	5.38 (2.58–11.23)	<0.001	5.21 (2.46–11.04)	<0.001	0.001
Any safety event, n (%)	0 (0.0)	1 (1.4)	−0.01 (−0.04 to 0.01)	0.33 (0.01–8.03)	N/A	N/A	N/A	N/A	

Risk differences, risk ratios, and odds ratios (ORs) are presented with 95% confidence intervals (CIs). Unadjusted ORs were estimated using univariable logistic regression models. Adjusted ORs were estimated using multivariable logistic regression including the TyG index, which remained slightly imbalanced after propensity score matching. Other matching covariates were well balanced and therefore were not included in the adjusted model. To account for multiple comparisons across outcome measures, *P* values from the multivariable models were adjusted using the Benjamini–Hochberg false discovery rate (FDR) method. A two-sided *p* < 0.05 was considered statistically significant. CI, confidence interval; DAPT, dual antiplatelet therapy; END, early neurological deterioration; ENI, early neurological improvement; mRS, modified Rankin Scale; OR, odds ratio.

### Secondary outcomes

**Figure 2:**
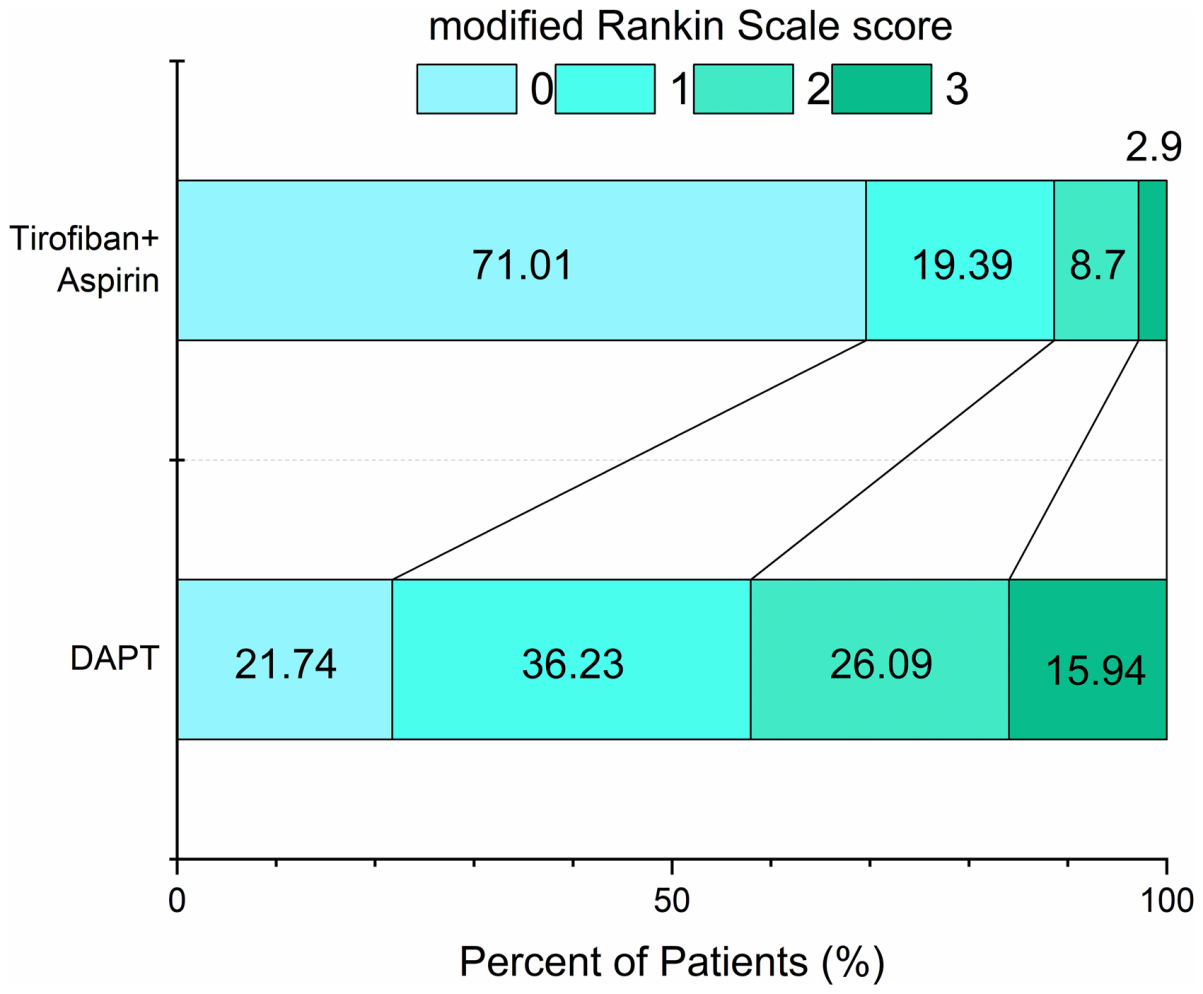
Distribution of 90-day modified Rankin Scale scores in the matched cohort. The distribution of 90-day modified Rankin Scale (mRS) scores is shown for patients in the propensity score–matched cohort. DAPT, dual antiplatelet therapy; mRS, modified Rankin Scale. Scores range from 0 (no symptoms) to 6 (death).

The distribution of 90-day mRS scores differed significantly between treatment groups, with a clear shift toward better functional outcomes in the tirofiban plus aspirin group. Ordinal logistic regression confirmed this shift, yielding an adjusted common odds ratio of 0.14 (95% CI, 0.07–0.29; *p* < 0.001) (Figure 2).

Favorable functional outcomes (mRS 0–2) at 90 days were observed in 97.1% of patients in the tirofiban plus aspirin group, compared with 84.1% in the DAPT group. After adjustment, tirofiban plus aspirin was associated with a higher probability of achieving mRS 0–2 (adjusted OR, 6.50; 95% CI, 1.34–31.45; *p* = 0.020).

**Figure 3:**
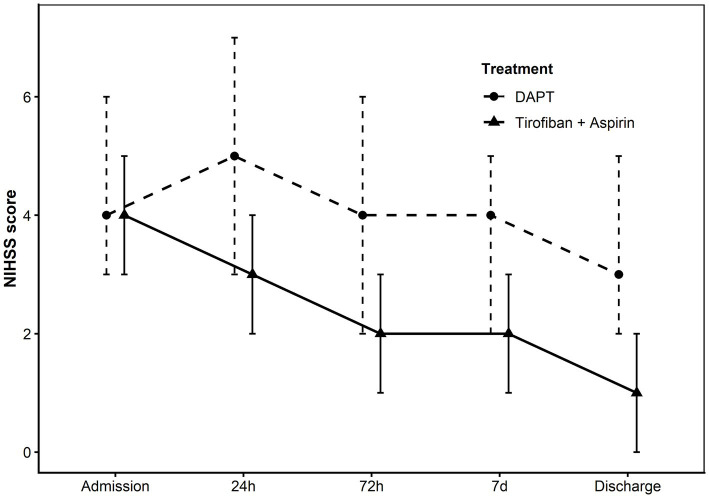
Dynamic changes in NIHSS scores during hospitalization in the propensity score–matched population. NIHSS scores are presented as median with interquartile range (IQR) for the tirofiban plus aspirin group and the dual antiplatelet therapy (DAPT) group at admission, 24 h, 72 h, 7 days, and at hospital discharge. DAPT, dual antiplatelet therapy; IQR, interquartile range; NIHSS, National Institutes of Health Stroke Scale.

Early neurological improvement occurred more frequently in the tirofiban plus aspirin group than in the DAPT group (75.4% vs. 36.2%). This association remained significant after adjustment for covariates (adjusted OR, 5.21; 95% CI, 2.46–11.04; *p* < 0.001). Patients receiving tirofiban plus aspirin also demonstrated greater reductions in NIHSS scores from baseline to hospital discharge, with a large effect size (partial η^2^ = 0.253). This benefit persisted throughout hospitalization (Figure 3).

### Sensitivity analyses

Sensitivity analyses varying caliper width and matching ratio yielded results consistent with the primary analysis. Across all scenarios, tirofiban plus aspirin remained associated with a lower risk of END and higher probability of achieving excellent functional outcome at 90 days, supporting the robustness of the main findings (Supplementary Table S8).

In an additional sensitivity analysis incorporating ln(SII) into the propensity score model, 60 matched pairs were successfully generated (Supplementary Table S1). The treatment effects remained consistent with the primary analysis: END occurred in 11.7% of patients in the T + A group versus 53.3% in the DAPT group (adjusted OR 0.055; 95% CI 0.015–0.163), and excellent functional outcomes were achieved in 91.7% versus 58.3% (adjusted OR 8.282; 95% CI 3.001–27.455) (Supplementary Table S2). This sensitivity analysis confirmed that the observed protective effect of tirofiban plus aspirin remained robust, supporting the notion that systemic inflammation is an intermediate factor rather than a confounder in treatment allocation.

### Safety outcomes

Safety events were infrequent in both treatment groups. No cases of symptomatic intracranial hemorrhage or major bleeding were observed in the tirofiban plus aspirin group. One patient (1.4%) in the DAPT group developed a punctate intracranial hemorrhage. No moderate or severe bleeding events occurred in either group (Table 2). Given the low event rates and the modest sample size, the study was underpowered to detect rare safety outcomes, such as symptomatic intracranial hemorrhage.

### Rescue therapy subgroup analysis

Among patients in the DAPT group who developed early neurological deterioration (*n* = 38), 26 received tirofiban as rescue therapy. At 90 days, excellent functional outcomes (mRS 0–1) were achieved in 65.4% of patients who received rescue tirofiban therapy, compared with 16.7% of those who continued DAPT alone.

Firth’s penalized logistic regression demonstrated a significantly higher likelihood of favorable functional outcomes associated with rescue tirofiban therapy (OR, 9.00; 95% CI, 1.41–99.71; *p* = 0.020). Ordinal logistic regression further showed a significant shift toward better functional outcomes in the rescue therapy subgroup (common OR, 0.09; 95% CI, 0.02–0.42; *p* = 0.002) (Table 3).

**Table 3:** Ninety-day functional outcomes in patients with END receiving rescue tirofiban therapy versus continued DAPT.

Outcomes	Rescue tirofiban (*n* = 26)	Continued DAPT (*n* = 12)	Risk difference (95% CI)	Risk ratio (95% CI)	OR (95% CI)	*P*
Excellent functional outcome (mRS score 0–1) at 90 d, n (%)	17 (65.4)	2 (16.7)	48.70 (20.80–76.60)	3.92 (1.15–13.36)	9.44 (1.69–52.73)	0.010
Favorable functional outcome (mRS score 0–2) at 90 d, n (%)	25 (96.2)	8 (66.7)	29.50 (6.10–52.90)	1.44 (1.01–2.06)	9.00 (1.41–99.71)	0.020
mRS score distribution at 90 d, median (IQR)	1(0.2)	2 (2.3)	N/A	N/A	0.09 (0.02–0.42)	0.002

Given the small sample size, Firth-penalized logistic regression was applied to reduce small-sample bias. Risk differences, risk ratios, and odds ratios (ORs) are presented with 95% confidence intervals (CIs). A two-sided *P* value <0.05 was considered statistically significant. CI, confidence interval; DAPT, dual antiplatelet therapy; END, early neurological deterioration; mRS, modified Rankin Scale.

### Subgroup analyses

**Figure 4:**
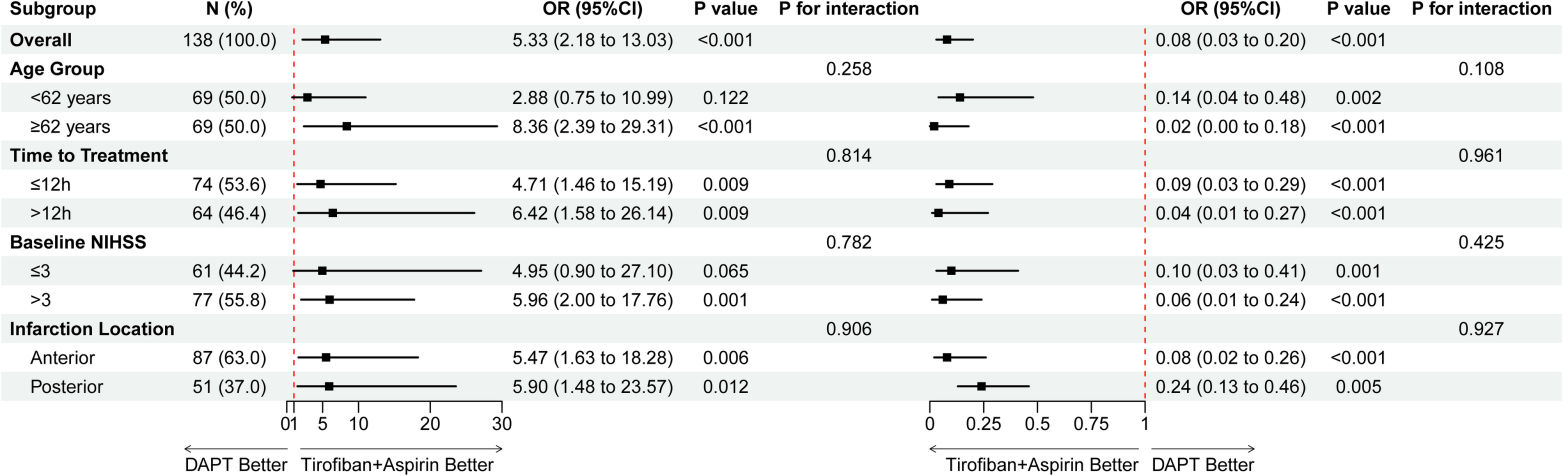
Forest plot of subgroup analyses for the effects of tirofiban plus aspirin versus dual antiplatelet therapy on the dual primary outcomes. Odds ratios (ORs) with 95% confidence intervals (CIs) are presented for prespecified subgroups, including age, onset-to-treatment time, baseline NIHSS score, and infarction location. Subgroup effects were assessed using tests for interaction. DAPT, dual antiplatelet therapy; END, early neurological deterioration; NIHSS, National Institutes of Health Stroke Scale; mRS, modified Rankin Scale; OR, odds ratio.

Prespecified subgroup analyses indicated that the effects of tirofiban plus aspirin on reducing early neurological deterioration and improving excellent functional outcomes were generally consistent across clinically relevant subgroups, including age, onset-to-treatment time, baseline NIHSS score, and infarct location. No statistically significant interactions were detected (all P for interaction > 0.05) (Figure 4).

In addition, baseline NIHSS score ≤3 was independently associated with both excellent and favorable functional outcomes at 90 days, whereas age ≤62 years was independently associated with excellent functional outcomes.

## Discussion

In this propensity score–matched cohort of patients with branch atheromatous disease, early treatment with tirofiban combined with aspirin was associated with a markedly lower incidence of early neurological deterioration and better functional outcomes at 90 days compared with standard dual antiplatelet therapy. The magnitude of the observed treatment effects should be interpreted cautiously given the retrospective design and potential residual confounding. Furthermore, among patients who experienced END despite initial DAPT, escalation to tirofiban as rescue therapy was associated with improved neurological recovery without an apparent increase in hemorrhagic complications. Collectively, these findings suggest that tirofiban may provide both preventive and rescue benefits in BAD. Nevertheless, given the observational design and the magnitude of the observed treatment effects, residual confounding cannot be entirely excluded despite careful propensity score matching. Importantly, the associations remained statistically significant after correction for multiple comparisons using the false discovery rate method, further supporting the robustness of the findings.

Early neurological deterioration in BAD is widely believed to result from progressive thrombus formation at the origins of perforating arteries affected by atherosclerotic plaques in the parent vessel (26). This process leads to stepwise infarct extension along the perforator territory rather than abrupt occlusion. Previous imaging studies have shown that infarct enlargement along the course of perforating arteries and increasing lesion burden are closely associated with neurological worsening (17, 27). In parallel, perfusion imaging has demonstrated that patients with BAD who develop END often exhibit reduced cerebral blood flow and limited collateral compensation, further aggravating ischemic injury (28, 29).

The pharmacological characteristics of tirofiban are consistent with these pathophysiological features. As a fast-acting and reversible glycoprotein IIb/IIIa receptor antagonist, tirofiban inhibits the final common pathway of platelet aggregation and produces immediate platelet inhibition (30). In contrast, oral P2Y12 inhibitors require metabolic activation and have a delayed onset of action (31). Rapid platelet inhibition during the early ischemic phase may therefore help limit thrombus propagation at perforator origins and stabilize microvascular perfusion. In addition, the short half-life of tirofiban allows prompt reversal of its antiplatelet effect, which may partially mitigate bleeding risk once the acute phase has passed (23).

Compared with other intensified antithrombotic strategies, such as argatroban combined with DAPT (10), the tirofiban-plus-aspirin regimen was associated with more favorable clinical outcomes in our cohort. This observation is biologically plausible, as BAD is characterized by platelet-rich thrombus formation rather than fibrin-dominant occlusion. Consequently, direct inhibition of platelet aggregation may represent a more pathophysiologically targeted approach than anticoagulation-based strategies in this setting.

Although tirofiban has been investigated in broader populations with acute ischemic stroke, including patients ineligible for reperfusion therapy and those with neurological worsening after thrombolysis (14–16), evidence specifically focused on BAD remains limited. To our knowledge, this study is among the first to restrict inclusion to radiologically defined BAD and to demonstrate both preventive and rescue effects of tirofiban in BAD-related END. The apparent benefit observed in the rescue therapy subgroup raises the possibility that tirofiban may attenuate ongoing microvascular compromise after clinical deterioration has occurred. This hypothesis is supported indirectly by cardiovascular studies showing improved microcirculatory perfusion with glycoprotein IIb/IIIa inhibition (32, 33). However, this mechanism remains speculative in the context of BAD and requires confirmation in prospective studies incorporating perfusion imaging. Moreover, the post-hoc nature of the rescue analysis introduces potential indication bias and immortal time bias, as treatment escalation was guided by clinical judgment after END had already developed.

Subgroup analyses showed that the beneficial effects of tirofiban were consistent across anterior and posterior circulation BAD. This finding differs from an earlier report in small vessel occlusion suggesting preferential benefit in anterior circulation strokes (34). One possible explanation is that previous studies included heterogeneous posterior circulation infarcts with embolic or large-artery etiologies. By limiting enrollment to imaging-confirmed BAD, our study reduced etiologic heterogeneity and may provide a more accurate estimate of treatment effects across vascular territories.

Although tirofiban improves overall outcomes in BAD, a subset of patients still experience END, indicating mechanisms beyond thrombus formation. Increasing evidence implicates inflammation in BAD-related END (35, 36), with platelets acting at the interface of thrombosis and immune activation (37). Importantly, Yang et al. reported that tirofiban does not significantly reduce systemic inflammatory markers, suggesting its benefit is largely antiplatelet (34). Consistently, our exploratory analyses showed that ln(SII) was independently associated with END, highlighting the contribution of systemic inflammatory burden. These observations suggest that incorporating anti-inflammatory strategies could complement antiplatelet therapy for high-risk patients, though further studies are needed to confirm this hypothesis.

Safety remains a critical concern when intensifying antiplatelet therapy. Although no symptomatic intracranial hemorrhage was observed in the present cohort, bleeding complications have been reported in other studies involving tirofiban (16, 38). It is important to note that, owing to the limited sample size, the study was underpowered to detect rare events such as symptomatic intracranial hemorrhage, and the absence of observed bleeding should not be interpreted as definitive evidence of safety. Given the retrospective design and limited sample size, particularly in the rescue subgroup, the safety findings should be interpreted cautiously. Larger prospective studies are needed to more precisely define the hemorrhagic risk associated with tirofiban-based strategies in BAD.

Despite propensity score matching, the TyG index, a marker of insulin resistance and metabolic dysregulation, remained imbalanced between groups after matching (SMD = 0.46). Although we adjusted for TyG in the multivariable outcome models, residual confounding by unmeasured metabolic factors cannot be entirely excluded. The TyG index has been associated with pro-inflammatory and pro-thrombotic states, which may influence both the risk of END and the response to antiplatelet therapy (39, 40). While sensitivity analyses confirmed that the treatment effects were consistent with and without TyG adjustment, the observed imbalance highlights the potential for residual metabolic confounding. Future studies incorporating comprehensive metabolic profiling (e.g., HbA1c, insulin levels, inflammatory cytokines) are warranted to further disentangle the interplay between metabolic status and treatment outcomes in BAD.

Importantly, many patients in our cohort achieved favorable outcomes with standard DAPT alone, and mild baseline neurological deficits were strongly associated with good prognosis. These findings suggest that routine upfront use of tirofiban in all patients with BAD may be unnecessary. Instead, a precision-treatment approach may be more appropriate, reserving early tirofiban for patients at high risk of END while allowing timely rescue therapy for those who deteriorate despite standard treatment.

Future research should focus on developing early risk stratification models for END that integrate clinical severity, imaging characteristics, and inflammatory biomarkers. Such tools may facilitate individualized treatment selection and help optimize the balance between efficacy and safety in the management of BAD.

Several limitations should be acknowledged. First, the retrospective design introduces the possibility of residual confounding despite propensity score matching. In particular, the imbalance in the TyG index after matching suggests potential residual metabolic confounding, which may have influenced the results even after statistical adjustment. Second, the single-center setting and modest sample size may limit generalizability. Moreover, our findings are limited to a single-center cohort of Chinese patients with acute, mild-to-moderate BAD (NIHSS < 15) and may not be generalizable to Western populations, elderly patients, or those with severe BAD. Third, the absence of high-resolution vessel wall imaging and perfusion data constrained mechanistic interpretation. Specifically, high-resolution vessel wall imaging was not routinely available, limiting the ability to directly assess parent artery plaque characteristics and the pathophysiology of END in BAD. Finally, patients with severe renal dysfunction were excluded, and no dose adjustment was applied, limiting extrapolation to populations with impaired tirofiban clearance (41). Prospective, multicenter studies with comprehensive imaging are required to validate these findings and refine patient selection.

It should also be noted that clopidogrel was initiated without a loading dose in the DAPT group during the study period, reflecting routine institutional practice for BAD management. This differs from the protocols used in large randomized trials of minor stroke and TIA, such as the CHANCE and POINT trials (42, 43), in which a clopidogrel loading dose was administered. The absence of a loading dose may theoretically delay the onset of platelet inhibition and could potentially influence early treatment effects. However, this regimen reflects real-world clinical practice in some centers managing BAD and therefore may enhance the pragmatic relevance of the present findings.

## Conclusion

In this single-center cohort of Chinese patients with acute, mild-to-moderate BAD (NIHSS < 15), early initiation of tirofiban plus aspirin was associated with reduced END and improved 90-day functional outcomes. However, given the single-center design and restricted patient population, these findings cannot be generalized to Western populations, elderly patients, or those with severe BAD. The efficacy and safety of tirofiban as a rescue or preventive therapy require validation in prospective, randomized studies before broader adoption.

## Data Availability

The raw data supporting the conclusions of this article will be made available by the authors, without undue reservation.
