# Supplementary material for: Tirofiban combined with aspirin for branch atheromatous disease: a propensity score–matched study
Source: Front Neurol. 2026 Apr 1;17:1802990. doi: 10.3389/fneur.2026.1802990 (PMC13079176; doi:10.3389/fneur.2026.1802990)
Supplement: Supplementary file 1 [file Table_1.docx]

**Supplementary Table S1.** Baseline characteristics and covariate balance before and after propensity score matching including ln(SII)

| **Variable** | **Before PSM (SMD)** | **After PSM (SMD)** |
| --- | --- | --- |
| Age | 0.082 | 0.003 |
| Sex | 0.112 | 0.071 |
| Onset-to-treatment time (OTT) | 0.045 | 0.027 |
| NIHSS score | 0.089 | 0.015 |
| Baseline mRS | 0.072 | 0.026 |
| ln(SII) | 0.155 | 0.026 |

**Footnotes:** Standardized mean difference (SMD) values <0.10 indicate adequate covariate balance. This sensitivity analysis incorporated ln(SII) into the propensity score model to evaluate the robustness of the primary findings. After matching, 60 pairs (120 patients) were successfully generated, with all covariates achieving adequate balance (SMD <0.10).

**Abbreviations:** SMD: standardized mean difference; NIHSS: National Institutes of Health Stroke Scale; mRS: modified Rankin Scale; ln(SII):natural logarithm of systemic immune-inflammation index.

**Supplementary Table S2.** Sensitivity analysis including ln(SII) in the propensity score–matched cohort (n = 120)

| **Outcome** | **T+A (n = 60)** | **DAPT (n = 60)** | **Unadjusted OR (95% CI)** | **Adjusted OR† (95% CI)** |
| --- | --- | --- | --- | --- |
| Early neurological deterioration (END) | 7 (11.7%) | 32 (53.3%) | 0.116 (0.042–0.281) | 0.055 (0.015–0.163) |
| 90-day mRS 0–1 | 55 (91.7%) | 35 (58.3%) | 7.857 (2.952–25.017) | 8.282 (3.001–27.455) |
| 90-day mRS 0–2 | 59 (98.3%) | 50 (83.3%) | 11.800 (2.150–220.300) | 14.750 (2.480–288.900) |

**Footnotes:** Adjusted OR† was derived from multivariable logistic regression using the same covariates as in the primary analysis, including the TyG index. Wide confidence intervals for mRS 0–2 reflect the small number of patients not achieving favorable outcomes in this matched cohort. This sensitivity analysis confirms that inclusion of ln(SII) in the propensity score model did not materially alter the estimated treatment effects for END or 90-day functional outcomes.

**Abbreviations:** OR: odds ratio; CI: confidence interval; mRS : modified Rankin Scale; ln(SII) : natural logarithm of systemic immune-inflammation index.

**Supplementary Table S3**. Baseline characteristics of unmatched patients after propensity score matching

| Characteristics | T + A (n=16) | DAPT (n=103) | p |
| --- | --- | --- | --- |
| Variables Used for PSM |  |  |  |
| Age, y; median (IQR) | 63 (54.5,75) | 65 (56,75) | 0.445 |
| Male, n (%) | 16 (100) | 55 (53.4) | 0.854 |
| Baseline NIHSS score, median (IQR) | 7 (5.5,8.5) | 3 (2,4) | <0.001 |
| Baseline mRS score, median (IQR) | 4 (3,4) | 2 (2,3) | <0.001 |
| Onset-to-treatment time, h; median (IQR) | 11.5 (8,19) | 24 (15.5,32.75) | 0.003 |
| Other Clinical Characteristics |  |  |  |
| Hypertension, n (%) | 4 (25) | 25 (24.3) | 0.950 |
| Diabetes, n (%) | 12 (75) | 82 (79.6) | 0.674 |
| Coronary artery disease, n (%) | 15 (93.8) | 98 (95.1) | 0.812 |
| History of stroke, n (%) | 12 (75) | 88 (85.4) | 0.289 |
| Smoking, n (%) | 8 (50.0) | 76 (73.8) | 0.052 |
| Drinking, n (%) | 12 (75.0) | 82 (79.6) | 0.674 |
| Infarct location, n (%) |  |  | 0.793 |
| Anterior circulation | 12 (75.0) | 74 (71.8) |  |
| Posterior circulation | 4 (25.0) | 29 (28.2) |  |
| Maximum infarct area, mm²; median (IQR) | 106.5 (44,127.5) | 63 (37.5,99) | 0.094 |
| Maximum infarct diameter, mm; median (IQR) | 13.2 (8.35,19) | 10.2 (7.25,14.1) | 0.116 |
| Number of involved slices; median (IQR) | 2 (1.5,3) | 2 (1,3) | 0.581 |
| Systolic BP at admission, mmHg; mean ± SD | 160.5 (154,173.5) | 152 (140,171) | 0.952 |
| Diastolic BP at admission, mmHg; median (IQR) | 91.63±13.44 | 89.12±12.04 | 0.447 |
| Laboratory examination |  |  |  |
| Ln(SII); median (IQR) | 6.56 (6.35,7.43) | 6.09 (5.78,6.48) | <0.001 |
| TyG index; median (IQR) | 1.64 (1.01,2.18) | 1.36 (0.96,1.82) | 0.459 |
| TC, mmol/L; mean ± SD | 5.04±1.23 | 5.08±1.22 | 0.918 |
| HDL, mmol/L; median (IQR) | 1.31 (1.03,1.65) | 1.26 (1.10,1.50) | 0.879 |
| LDL, mmol/L; median (IQR) | 3.06 (1.93,3.47) | 2.91 (2.36,3.66) | 0.586 |

**Footnotes:** Continuous variables are presented as median (IQR) or mean ± SD, as appropriate. Categorical variables are presented as n (%).Comparisons between groups were performed using Mann–Whitney U test for non-normally distributed continuous variables, t-test for normally distributed continuous variables, and chi-square or Fisher’s exact test for categorical variables. T + A: tirofiban plus aspirin; DAPT: dual antiplatelet therapy (aspirin plus clopidogrel).This table shows baseline characteristics of patients who were not successfully matched in the propensity score matching process. It is provided to illustrate the characteristics of unmatched patients and inform the matching process.

**Supplementary Table S4**. Baseline Characteristics of Matched and Unmatched Cohorts

| **Variable** | **Matched (n=138)** | **Unmatched (n=119)** | **\|SMD\|** |
| --- | --- | --- | --- |
| Age | 63.45 | 65.03 | 0.141 |
| Male | 0.688 | 0.597 | 0.192 |
| Baseline NIHSS score | 4.41 | 3.95 | 0.190 |
| Baseline mRS score | 2.97 | 2.59 | 0.333 |
| Onset-to-treatment time | 15.58 | 24.27 | 0.630 |
| Smoking | 0.464 | 0.294 | 0.355 |
| Drinking | 0.290 | 0.210 | 0.185 |
| Hypertension | 0.696 | 0.756 | 0.136 |
| Diabetes | 0.188 | 0.210 | 0.054 |
| Coronary artery disease | 0.058 | 0.050 | 0.033 |
| History of stroke | 0.116 | 0.160 | 0.127 |
| Systolic BP | 155.99 | 156.69 | 0.032 |
| Diastolic BP | 90.28 | 89.45 | 0.065 |
| Infarct location | 0.732 | 0.723 | 0.021 |
| Maximum infarct area | 0.86 | 0.76 | 0.112 |
| Maximum infarct diameter | 1.16 | 1.19 | 0.044 |
| Number of involved slices | 2.30 | 2.20 | 0.090 |
| Ln(SII) | 6.35 | 6.28 | 0.099 |
| TyG index | 1.69 | 1.49 | 0.273 |
| HDL | 1.25 | 1.38 | 0.317 |
| LDL | 2.86 | 2.98 | 0.124 |
| TC | 4.86 | 5.07 | 0.183 |

**Footnotes:** Continuous variables are presented as mean or median, as appropriate; categorical variables are presented as proportions. SMD: standardized mean difference, used to assess balance between matched and unmatched patients; values <0.1 are generally considered negligible. Matched: patients successfully included in the propensity score–matched cohort (n=138). Unmatched: patients not successfully matched after propensity score matching (n=119). This table illustrates the baseline characteristics of matched and unmatched patients to evaluate the effectiveness of the matching procedure.

**Abbreviations:** T + A, tirofiban plus aspirin; DAPT, dual antiplatelet therapy (aspirin plus clopidogrel); mRS, modified Rankin Scale; NIHSS, National Institutes of Health Stroke Scale; BP, blood pressure; HDL, high-density lipoprotein; LDL, low-density lipoprotein; TC, total cholesterol; TyG, triglyceride–glucose index; SII, systemic immune-inflammation index.

**Supplementary Table S5**. Baseline clinical and laboratory characteristics of patients with branch atheromatous disease before propensity score matching

| **Characteristics** | T+A | DAPT | P value |
| --- | --- | --- | --- |
| **Variables Used for PSM** |  |  |  |
| Age, y; median (IQR) | 60 (55.5 to 72.5) | 64 (56 to 75) | 0.279 |
| Male, n (%) | 62 (73.8) | 104 (60.1) |  |
| Baseline NIHSS score, median (IQR) | 4 (3,6.5) | 3 (2,5) | 0.004 |
| Baseline mRS score, median (IQR) | 4 (2,4) | 3 (2,4) | <0.001 |
| Onset-to-treatment time, h; median (IQR) | 10 (6,24) | 22.5 (11 to 27) | <0.001 |
| **Other Clinical Characteristics** |  |  |  |
| Hypertension, n (%) | 61 (72.6) | 125 (72.3) | 0.951 |
| Diabetes, n (%) | 18 (21.4) | 33 (19.1) | 0.657 |
| Coronary artery disease, n (%) | 2 (2.4) | 12 (6.9) | 0.131 |
| History of stroke, n (%) | 10 (11.9) | 25 (14.5) | 0.577 |
| Smoking, n (%) | 39 (46.4) | 60 (34.7) | 0.070 |
| Drinking, n (%) | 27 (32.1) | 38 (22.0) | 0.078 |
| Infarct location, n (%) |  |  | 0.351 |
| Anterior circulation | 58 (69.0) | 129 (74.6) |  |
| Posterior circulation | 26 (31.0) | 44 (25.4) |  |
| Maximum infarct area, mm²; median (IQR) | 64 (38,111) | 63 (31,150) | 0.327 |
| Maximum infarct diameter, mm; median (IQR) | 11 (8,15) | 11 (7.2,14.8) | 0.317 |
| Number of involved slices; median (IQR) | 2 (2,3) | 2 (1,3) | 0.630 |
| Systolic BP at admission, mmHg; median (IQR) | 158 (140 to 171.5) | 152 (140 to 171) | 0.489 |
| Diastolic BP at admission, mmHg; median (IQR) | 90 (80 to 101) | 88 (80 to 98.5) | 0.364 |
| **Laboratory examination** |  |  |  |
| Ln(SII); median (IQR) | 6.41 (6.15 to 6.88) | 6.11 (5.84 to 6.69) | 0.002 |
| TyG index; median (IQR) | 1.83 (1.08 to 2.29) | 1.39 (1.00 to 1.83) | 0.001 |
| TC, mmol/L; median (IQR) | 5.00 (4.10 to 5.60) | 4.90 (4.20 to 5.75) | 0.989 |
| HDL, mmol/L; median (IQR) | 1.20 (1.05 to 1.40) | 1.24 (1.07 to 1.50) | 0.354 |
| LDL, mmol/L; median (IQR) | 2.75 (1.98 to 3.39) | 2.91 (2.35 to 3.55) | 0.138 |

**Footnotes:**
Continuous variables are presented as median (interquartile range, IQR), and categorical variables are presented as number (percentage).
Variables listed under “Variables used for PSM” were included in the propensity score matching model.
P values were calculated using the Mann–Whitney U test for continuous variables and the chi-square test or Fisher’s exact test for categorical variables, as appropriate.
Systolic and diastolic blood pressure were measured at hospital admission.

**Abbreviations:** T+A, tirofiban plus aspirin; DAPT, dual antiplatelet therapy; NIHSS, National Institutes of Health Stroke Scale; mRS, modified Rankin Scale; SII, systemic immune-inflammation index; ln(SII), natural logarithm of SII; TyG index, triglyceride–glucose index; TC, total cholesterol; HDL-C, high-density lipoprotein cholesterol; LDL-C, low-density lipoprotein cholesterol; BP, blood pressure.

**Supplementary Table S6**：Outcomes in the overall cohort

| Outcomes | T+A | DAPT | OR (95%Cl) | P value |
| --- | --- | --- | --- | --- |
| Early neurological deterioration (END) | 11 (13.1) | 82 (47.4) | 0.10 (0.05 to 0.24) | <0.001 |
| Excellent functional outcome (mRS 0–1) at 90 days | 72（85.7） | 94 (54.3) | 13.82 (5.65 to 33.82) | <0.001 |
| Favorable functonal outcome (mRS 0–2) at 90 days | 81 (96.4) | 136 (78.6) | 18.01 (4.43 to 73.24) | <0.001 |
| Early neurological improvement (ENI) | 63 (75) | 44 (25.4) | 11.99 (5.57 to 25.79) | <0.001 |

**Footnotes:**
Data are presented as number (percentage).
Odds ratios (ORs) with 95% confidence intervals (CIs) were calculated using univariable logistic regression to compare outcomes between treatment groups.
These analyses were performed in the unmatched cohort and were not adjusted for baseline imbalances; therefore, the results should be interpreted with caution.

**Abbreviations:** T+A, tirofiban plus aspirin; DAPT, dual antiplatelet therapy; END, early neurological deterioration; mRS, modified Rankin Scale; ENI, early neurological improvement; OR, odds ratio; CI, confidence interval.

**Supplementary Table** **S7**. Multivariable logistic regression analysis of factors associated with early neurological deterioration in the overall cohort

| Variable | Adjusted OR (95% CI) | P value |
| --- | --- | --- |
| Treatment (T+A vs DAPT) | 0.08 (0.04 to 0.19) | <0.001 |
| ln(SII) | 2.41 (1.48 to 3.92) | <0.001 |
| Onset-to-treatment time | 0.97 (0.95 to 1.00) | 0.017 |
| Baseline NIHSS | 0.85 (0.71 to 1.01) | 0.069 |
| Baseline mRS | 1.29 (0.91 to 1.82) | 0.158 |
| TyG | 1.31 (0.67 to 2.55) | 0.433 |

**Footnotes:**
Multivariable logistic regression analysis was performed to identify factors independently associated with early neurological deterioration (END) in the unmatched cohort.
Adjusted odds ratios (ORs) with 95% confidence intervals (CIs) are reported.
Variables were selected based on clinical relevance, prior literature, and overlap with variables included in the propensity score model.
A two-sided P value < 0.05 was considered statistically significant.

**Abbreviations:** T+A, tirofiban plus aspirin; DAPT, dual antiplatelet therapy; END, early neurological deterioration; ln(SII), natural logarithm of systemic immune-inflammation index; NIHSS, National Institutes of Health Stroke Scale; mRS, modified Rankin Scale; TyG index, triglyceride–glucose index; OR, odds ratio; CI, confidence interval.

**Supplementary Table S8**. Sensitivity analysis of propensity score–matched cohorts

| Caliper | Matching ratio | n_matched | OR_END_unadj (95% CI) | OR_END_adj (95% CI, adjusted for TyG) | OR_mRS_unadj (95% CI) | OR_mRS_adj (95% CI, adjusted for TyG) |
| --- | --- | --- | --- | --- | --- | --- |
| 0.01 | 1:1 | 76 | 0.34 (0.12–0.90) | 0.29 (0.10–0.80) | 4.96 (1.56–9.20) | 5.35 (1.62–17.75) |
| 0.02 | 1:1 | 106 | 0.28 (0.11–0.66) | 0.23 (0.08–0.55) | 5.04 (2.00–14.06) | 5.16 (2.02–14.67) |
| 0.03 | 1:1 | 126 | 0.24 (0.10–0.53) | 0.20 (0.08–0.47) | 5.81 (2.54–14.42) | 5.84 (2.53–14.66) |
| 0.02 | 1:2 | 124 | 0.25 (0.11–0.56) | 0.21 (0.08–0.49) | 4.81 (2.00–8.98) | 4.73 (1.95–12.85) |

Footnotes:

Sensitivity analyses of propensity score–matched cohorts using different caliper widths and matching ratios are presented.

OR_END_unadj and OR_mRS_unadj: unadjusted odds ratios for early neurological deterioration (END) and unfavorable 90-day functional outcome (mRS ≥3).

OR_END_adj and OR_mRS_adj: odds ratios adjusted for the TyG index, the only covariate that remained imbalanced after matching.

Caliper: width of the propensity score caliper used for matching.

Matching ratio: ratio of treated to control patients in each matched cohort.

n_matched: number of patients included in each matched cohort.

Abbreviations: END, early neurological deterioration; mRS, modified Rankin Scale; OR, odds ratio; CI, confidence interval; TyG, triglyceride–glucose index.

Supplementary Figure 1. Kernel density plots of propensity scores before and after matching


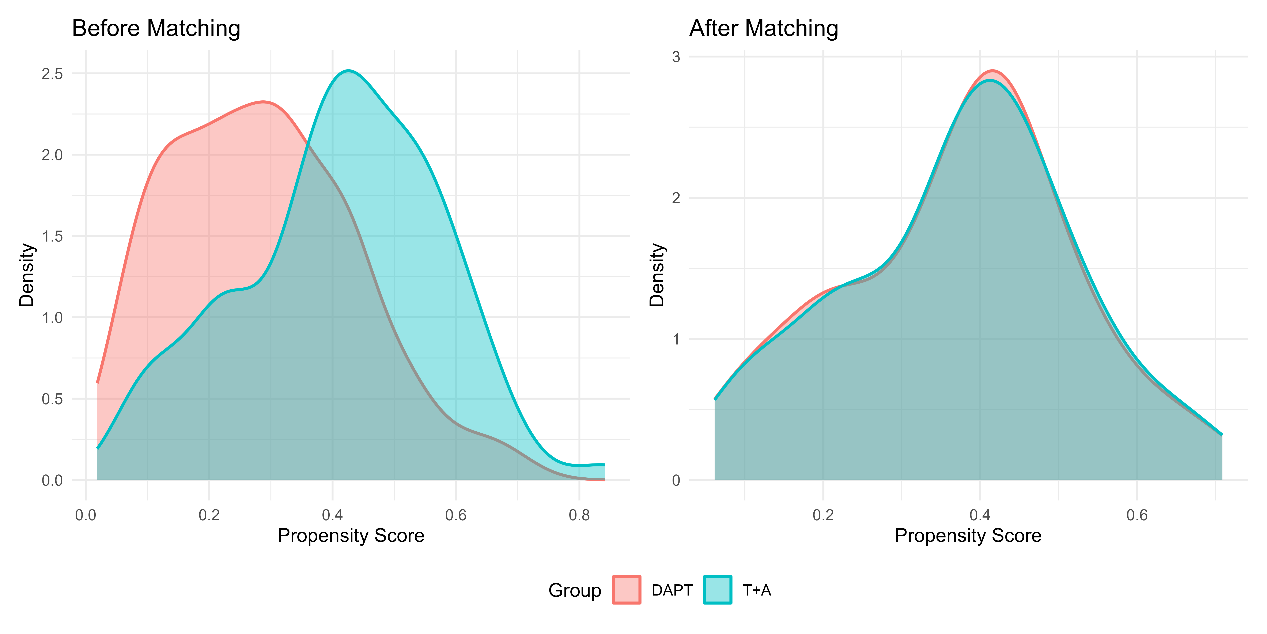


The plots show the distribution of propensity scores in the tirofiban plus aspirin (T+A) and dual antiplatelet therapy (DAPT) groups. Substantial overlap of the distributions after matching confirms adequate common support and successful balance of covariates between treatment groups.

Footnotes:T+A = tirofiban plus aspirin group; DAPT = dual antiplatelet therapy group.

Kernel density estimates were used to visualize the distribution of propensity scores.
